# High Dose Antipsychotic Therapy (HDAT) and Physical Health Monitoring for Patients Under the Liverpool Homeless Outreach Service

**DOI:** 10.1192/bjo.2025.10674

**Published:** 2025-06-20

**Authors:** Sibanda Shelton, Monira Sharif, Tom Ebbatson, Kauser Tabani

**Affiliations:** Mersey Care NHS Foundation Trust, Liverpool, United Kingdom

## Abstract

**Aims:** To assess the antipsychotic burden and adherence to the Trust policy on HDAT and physical health monitoring for patients under the Liverpool Homeless Service.

**Methods:** Patient records were assessed for a three-month period between July to September 2024 to look at the antipsychotic burden for patients under the Liverpool Homeless Service. Sources of information included patient electronic records, and General Practitioner summaries. A two-stage process was then carried out depending on the HDAT calculations. For patients found to be HDAT, records were checked to measure the adherence to the protocol. Trust Protocol would require bloods and ECG to be done, followed by repeat tests at 3 and 6 months. For the rest of the patients on the caseload we assessed whether physical health monitoring had been done per policy. Trust policy on this was yearly Body Mass Index, full blood count, Liver function tests, Renal profile, HBA1C level, lipid profile, serum prolactin. Compliance to these standards was set at 100%. A total of 40 patients were included in this audit

**Results:** We found that 2.5% of the patients in the service were receiving HDAT. HDAT protocols were not followed for these patients. With regards to physical health monitoring 62.5% of the patients had received the stipulated yearly bloods tests. 55% had Body Mass Index done. Reasons given for non-compliance to these checks included lack of engagement from service users, lack of timely reviews. 77.5% of patients assessed had ECG monitoring done, and this was on time in 60%.

**Conclusion:** Use of high-dose antipsychotics in the service was low, at 2.5%. There was low uptake of HDAT protocol in these patients. Physical health reviews were noted to be adherent to the policy in about half the caseload. To this end recommendations were made for a system to identify patients due for their physical health checks. Awareness was also to be raised in the team regarding HDAT.

